# Phosphorylation of RelA/p65 Ser536 inhibits the progression and metastasis of hepatocellular carcinoma by mediating cytoplasmic retention of NF-κB p65

**DOI:** 10.1093/gastro/goae094

**Published:** 2024-11-04

**Authors:** Wentao Zuo, Haoyang Ma, Jianghui Bi, Tiaolan Li, Yifeng Mo, Shiyu Yu, Jia Wang, Beiqing Li, Jinfeng Huang, Yongwen Li, Li Li

**Affiliations:** College of Basic Medical, Guilin Medical University, Guilin, Guangxi, P. R. China; College of Basic Medical, Guilin Medical University, Guilin, Guangxi, P. R. China; College of Pharmacy, Guilin Medical University, Guilin, Guangxi, P. R. China; College of Basic Medical, Guilin Medical University, Guilin, Guangxi, P. R. China; College of Pharmacy, Guilin Medical University, Guilin, Guangxi, P. R. China; College of Pharmacy, Guilin Medical University, Guilin, Guangxi, P. R. China; College of Pharmacy, Guilin Medical University, Guilin, Guangxi, P. R. China; College of Pharmacy, Guilin Medical University, Guilin, Guangxi, P. R. China; College of Pharmacy, Guilin Medical University, Guilin, Guangxi, P. R. China; College of Pharmacy, Guilin Medical University, Guilin, Guangxi, P. R. China; College of Basic Medical, Guilin Medical University, Guilin, Guangxi, P. R. China

**Keywords:** HCC, NF-κB, RelA/p65, Ser536 phosphorylation, apoptosis, metastasis

## Abstract

**Background:**

Intrahepatic and extrahepatic metastases contribute to the high recurrence rate and mortality of hepatocellular carcinoma (HCC). Constitutive activation of nuclear factor-κB (NF-κB) is a crucial feature of HCC. NF-κB p65 (p50–p65) is the most common dimeric form. Ser536 acts as an essential phosphorylation site of RelA/p65. However, the effect of RelA/p65 Ser536 phosphorylation on progression and metastases during intermediate and advanced HCC has not been reported.

**Methods:**

Phosphorylation of RelA/p65 (p-p65 Ser536) and NF-κB p65 were detected by using immunohistochemical staining in HCC tissue samples. The biological effects of RelA/p65 Ser536 phosphorylation were evaluated by using xenograft and metastasis models. NF-κB p65 nuclear translocation was detected by using Western blotting. The binding of NF-κB p65 to the *BCL2*, *SNAIL*, and *MMP9* promoters was detected by using chromatin immunoprecipitation. The biological effects on proliferation, migration, invasion, and epithelial–mesenchymal transition were assessed by using tetrazolium-based colorimetry, colony formation, EdU incorporation, flow cytometry, cell wound healing, and transwell assay.

**Results:**

NF-κB p65 is highly expressed, while p-p65 Ser536 is not well expressed in intermediate and advanced HCC tissues. *In vivo* experiments demonstrated that a phosphorylation-mimetic mutant of RelA/p65 Ser536 (p65/S536D) prevents tumor progression and metastasis. *In vitro* experiments showed that p65/S536D inhibits proliferation, migration, and invasion. Mechanistically, RelA/p65 Ser536 phosphorylation inhibits NF-κB p65 nuclear translocation and reduces NF-κB p65 binding to the *BCL2*, *SNAIL*, and *MMP9* promoters.

**Conclusions:**

RelA/p65 Ser536 phosphorylation was detrimental to NF-κB p65 entry into the nucleus and inhibited HCC progression and metastasis by reducing *BCL2*, *SNAIL*, and *MMP9*. The phosphorylation site of RelA/p65 Ser536 has excellent potential to be a promising target for NF-κB-targeted therapy in HCC.

## Introduction

Hepatocellular carcinoma (HCC) is the sixth-most common cancer and the third leading cause of cancer death worldwide [1, 2]. When HCC is diagnosed, the patient’s tumor is usually at an intermediate or advanced stage and the tumor cells have spread or metastasized to distant locations [3, 4]. Intrahepatic and extrahepatic metastases contribute to the high mortality and recurrence rates of HCC [5]. Therefore, inhibition of the metastasis of HCC is an effective strategy for treating intermediate and advanced HCC.

The main feature for distinguishing HCC cells from normal hepatocytes is the constitutive activation of nuclear factor-κB (NF-κB) [6]. The NF-κB family includes RelA (p65), RelB, c-Rel, NF-κB1 (p50/p105), and NF-κB2 (p52/p100) [7]. Of these, NF-κB p65 (p50–p65) is the most common dimeric form and is most abundant in cells [8]. RelA/p65 contains two potent *trans*-activating structural domains at its C-terminus. It possesses powerful transcriptional activity and is probably the most potent activator of most genes with κB sites [9]. NF-κB activation is tightly controlled and undergoes multiple posttranscriptional modifications, such as ubiquitination, acetylation, methylation, and phosphorylation [10]. In particular, posttranscriptional modifications of RelA/p65 play a key role in fine-tuning the transcriptional activity of NF-κB [10, 11]. Therefore, phosphorylation—a rapid and powerful mechanism to regulate NF-κB signaling pathway activation positively or negatively—has received the most attention [12].

For RelA/p65, 13 phosphosites have been identified [13, 14]. Ser536 is a vital phosphorylation site and can be phosphorylated by various kinases to respond to stimuli [15]. However, Ser536 presents two opposite states in tumor genesis and progression [16]. Xu *et al*. [17] reported increased expression of RelA/p65 Ser536 phosphorylation during acute inflammation-mediated hepatocarcinogenesis. In studies of andrographolide and magnolol inhibiting tumorigenesis and progression, RelA/p65 Ser536 phosphorylation was found to be downregulated [18, 19]. Controversially, there is increasing evidence that RelA/p65 Ser536 phosphorylation plays a negative regulatory role. Phosphorylation of RelA/p65 Ser536 promotes apoptosis in cervical, colon, breast, and prostate cancers [20, 21]. However, the role of RelA/p65 Ser536 phosphorylation in intermediate and advanced HCC has yet to be reported, drawing our group’s attention.

In this context, we first found that intermediate and advanced HCC with high NF-κB p65 expression but low p-p65 Ser536 expression is inversely correlated with HCC malignancy. Two p65 mutants were constructed to investigate the effect of RelA/p65 Ser536 phosphorylation on HCC. Specifically, the serine at the Ser536 site mutated to alanine and could not then be phosphorylated (p65/S536A). Moreover, the serine at the Ser536 site mutated to aspartate and then mimicked phosphorylation (p65/S536D). The results of animal and cellular experiments demonstrated that p65/S536D inhibits HCC progression and metastasis by blocking NF-κB p65 entry into the nucleus, inhibiting *BCL2*, *SNAIL*, and *MMP9*, and reversing the epithelial–mesenchymal transition (EMT). Our findings on the correlation between RelA/p65 Ser536 phosphorylation and HCC progression and metastasis are important for the precise treatment of middle- and late-stage HCC and provide a foundation for clinical treatment.

## Materials and methods

### Clinical specimens and immunohistochemical staining

HCC tissue samples were obtained from the Second Affiliated Hospital of Guilin Medical University. Tissue samples were rapidly stored in liquid nitrogen after separation from the human body. This research was authorized by Guilin Medical University’s Ethics Committee (No. GYLL2022017).

For IHC, tissue samples were fixed and paraffin-embedded, and 3-μm sections were stained. The sections were individually dewaxed under different ethanol concentrations and antigenic repair was performed by adding ethylene diamine tetraacetic acid antigen repair solution at 95°C. Then, endogenous peroxidase blockers were added according to the instructions (Cat#: DS-004; ZSGB Biotech, Beijing, China). The sections were incubated with anti-NF-κB p65 (1:200; ab86299; Abcam, Cambridge, UK) and anti-p-p65 Ser536 (1:200; SC-8008; Santa Cruz, CA, USA) overnight at 4°C. The next day, sections were incubated with secondary antibodies and stained with new fuchsin or DAB for 10 min at 37°C and hematoxylin for 30 s. The staining intensity was assessed by using the IHC Toolbox (NIH, USA). Average optical density (AOD) = integrated optical density (IOD)/area.

### Cell culture

HepG2, Huh7, SK-HEP-1, and SK-HEP-1-Luc2-tdT cells were obtained from the Cell Resource Center, Chinese Academy of Medical Sciences (Beijing, China). HCC cells were cultured in RPMI 1640 or DMEM (Gibco, Waltham, MA, USA) containing 10% fetal bovine serum (FBS; Gibco) with 1% penicillin–streptomycin (Solarbio, Beijing, China) in a cell culture incubator (37°C with 5% CO_2_).

### Transfection

The p65 shRNA plasmid (pSGU6-RFP-Neo-NF-κB p65) was obtained from Sangon Biotech Co., Ltd (Shanghai, China). p65 shRNA target sequences are available in Supplementary Table 1. The pCMV-RelA/p65-S536A (p65/S536A) and pCMV-RelA/p65-S536D (p65/S536D) plasmids were commercially purchased from Shanghai Outdo Biotech Co., Ltd (Shanghai, China). For p65/S536A (phosphorylation-deficient mutant), the amino acid sequence switches from serine to alanine at the Ser536 site and alanine cannot be phosphorylated. In contrast, for p65/S536D (a phosphorylation-mimetic mutant), the amino acid sequence switches from serine to aspartic acid at the Ser536 site, and the structure of aspartic acid is identical to that of phosphorylated serine. The p65 shRNA2 target sequence is AGG ACA TAT GAG ACC TTC AAG A. However, in mutants, this shRNA target region is mutated to AGA ACC TAC GAA ACA TTT AAA A, which confers resistance to p65 shRNA silencing under the condition of no change in the original amino acids. HCC cells were transfected with the p65 shRNA plasmid and then transferred into vector, p65/S536A, or p65/S536D for subsequent experiments.

### Tetrazolium-based colorimetry

HCC cells (6,000 cells/well) were cultured in 96-well plates. Total 20-μL tetrazolium-based colorimetry (MTT) (0.5 mg/mL; Cat#: M8180; Beyotime, Shanghai, China) was assigned and incubated for 4 h. The absorbance was detected by using a microplate reader (Infinite 200; Tecan, Switzerland).

### Colony formation

HCC cells (500 cells/well) were cultured in six-well plates. After 15 days, the HCC cells were rinsed, fixed, permeabilized, and stained. The number of colonies of cells was counted by using ImageJ software (NIH).

### EdU incorporation assay

HCC cells were transfected with the p65 shRNA plasmid without the RFP reporter gene and then transferred into vector, p65/S536A, or p65/S536D. HCC cells (1 × 10^5^ cells/well) were cultured in six-well plates. An equal volume of 20-μM EdU working solution was added according to the EdU assay kit (Cat#: K1076; Apexbio, Houston, Texas, USA) and incubated for 24 h. HCC cells were fixed, permeabilized, incubated with a click-reaction solution, and stained with Hoechest 33342. The nuclei of proliferating cells are stained red.

### Flow cytometry

HCC cells were transfected with the p65 shRNA plasmid without the RFP reporter gene and then transferred into vector, p65/S536A, or p65/S536D. HCC cells (1 × 10^6^ cells/well) were cultured in six-well plates for 48 h. HCC cells were collected and fixed overnight with 70% cold ethanol at 4°C. The next day, cells were stained with propidium iodide (PI) according to the Cell Cycle and Apoptosis Analysis Kit (Cat#: C1052; Beyotime). HCC cells were stained with APC-Annexin and PI according to the APC Annexin V Apoptosis Detection Kit with PI (Cat#: 640932; BioLegend, San Diego, CA, USA). HCC cell cycle distribution and apoptosis were analysed via flow cytometry (FACSCanto Plus; BD Biosciences, New Jersey, USA).

### Wound-healing assay

HCC cells (3 × 10^5^ cells/well) were incubated in 12-well plates and were scratched with a sterile pipette tip for 24 or 48 h. Cell wound healing was observed and photographed under an inverted microscope (HBO 100; Carl Zeiss, Oberkochen, GER).

### Migration and invasion assays

HCC cells (5 × 10^4^ cells) were added to the top chamber with Matrigel (Cat#: 0827045; ABWbio, Shanghai, China) and 20% FBS medium was added to the bottom chamber. After 24 h, the top chamber was rinsed, fixed, permeabilized, and stained. Finally, HCC cells were observed by using an inverted microscopy (HBO 100). ImageJ software (NIH) was used to count the number of cells that crossed the membrane.

### Western blot

Total protein was extracted from HCC cells with radio immunoprecipitation assay lysis buffer (Cat#: P0013B; Beyotime) and cytoplasmic and nuclear proteins were extracted from HCC cells by using a cytoplasmic protein/nuclear protein extraction kit (Cat#: C510001; Sangon). Equal quantities of proteins were separated by using sodium dodecyl sulfate–polyacrylamide gel electrophoresis and shifted to nitrocellulose membranes. Then, the nitrocellulose membranes were blocked, incubated with the corresponding antibodies (Supplementary Table 2), and then exposed to EZ ECL Pico Luminescent Liquid (Cat#: AP34L024; Life-iLab, Shanghai, China). The grayscale values were analysed by using a gel imaging system (Bio-Rad, California, USA).

### Reverse transcription–polymerase chain reaction

Total RNA was extracted from HCC cells by using a total RNA extraction kit (Cat#: DP424; Tiangen, Beijing, China) and reverse-transcribed into cDNA. The target gene was amplified and the primer sequence information is summarized in Supplementary Table 3. Subsequently, the amplified polymerase chain reaction (PCR) products were detected by using horizontal electrophoresis and the density was evaluated by using a gel imaging system (Bio-Rad).

### Chromatin immunoprecipitation

HCC cells (3 × 10^6^ cells) were cultivated in a dish for 48 h, fixed with 37% formaldehyde, and lysed. The chromatin was interrupted by using ultrasound. DNA was immunoprecipitated by using the anti-NF-κB p65 antibody (Cat#: 8242; Cell Signaling Technology, Inc, Danvers, MA, USA) according to the manufacturer’s recommendations (Cat#: P2078; Beyotime) and purified by using a DNA purification kit (Cat#: D0033; Beyotime). The immunoprecipitated DNA was analysed via reverse transcription (RT)-PCR. The primer sequences are shown in Supplementary Table 4.

### Animal experiments

Male BALB/c nude mice (5 weeks old, 18–20 g) were obtained from Hunan SJA Laboratory Animal Co., Ltd (Hunan, China), kept under standard conditions, and fed ad libitum. All animal experiments were authorized by Guilin Medical University's Ethics Committee (No. GLMC202303203). In xenograft models, nude mice were injected with SK-HEP-1 cells (5 × 10^6^ cells). After 3 days, the mice were randomly divided into vector, p65/S536A, and p65/S536D groups (*n *=* *6). AAV-vector (AAV-hTERT-EGFP), AAV-p65/S536A, or AAV-p65/S536D plasmid solution was injected into the tumors. The tumor volume was calculated by using the following formula: *V* = 0.5 × length × width^2^ [22]. After 21 days, the mice were anesthetized with 1.25% 2,2,2-tribromoethanol (0.02 mL/g; Cat#: M2910; Aibei Biotech, Nanjing, China) and sacrificed, and subcutaneous tumors were detected by using hemotoxylin and eosin staining and immunohistochemical staining.

In the lung metastasis model, SK-HEP-1-Luc2-tdT cells (1.5 × 10^6^ cells) mixed with AAV-vector, AAV-p65/S536A, or AAV-p65/S536D plasmid solution were injected into the tail veins of nude mice by using a sterile syringe (*n *=* *6). Tumor metastasis and growth were monitored by using an optical imaging system (Caliper Life Sciences, Hopkinton, USA) after anesthesia (0.02 mL/g; Cat#: M2910; Aibei Biotech) every 12 days. After 36 days of feeding and observation, the mice were sacrificed. All lungs were excised and fixed for further analysis.

### Statistical analysis

All data were analysed by using SPSS 26.0 (IBM, Armonk, NY, USA) and are presented as means ± standard deviation (SD). A one-way analysis of variance, Student's *t*-test and Kruskal–Wallis H test were used to analyse and calculate the data. *P *<* *0.05 was considered statistically significant.

## Results

### The expression of RelA/p65 Ser536 phosphorylation is low in HCC

**Figure 1.**
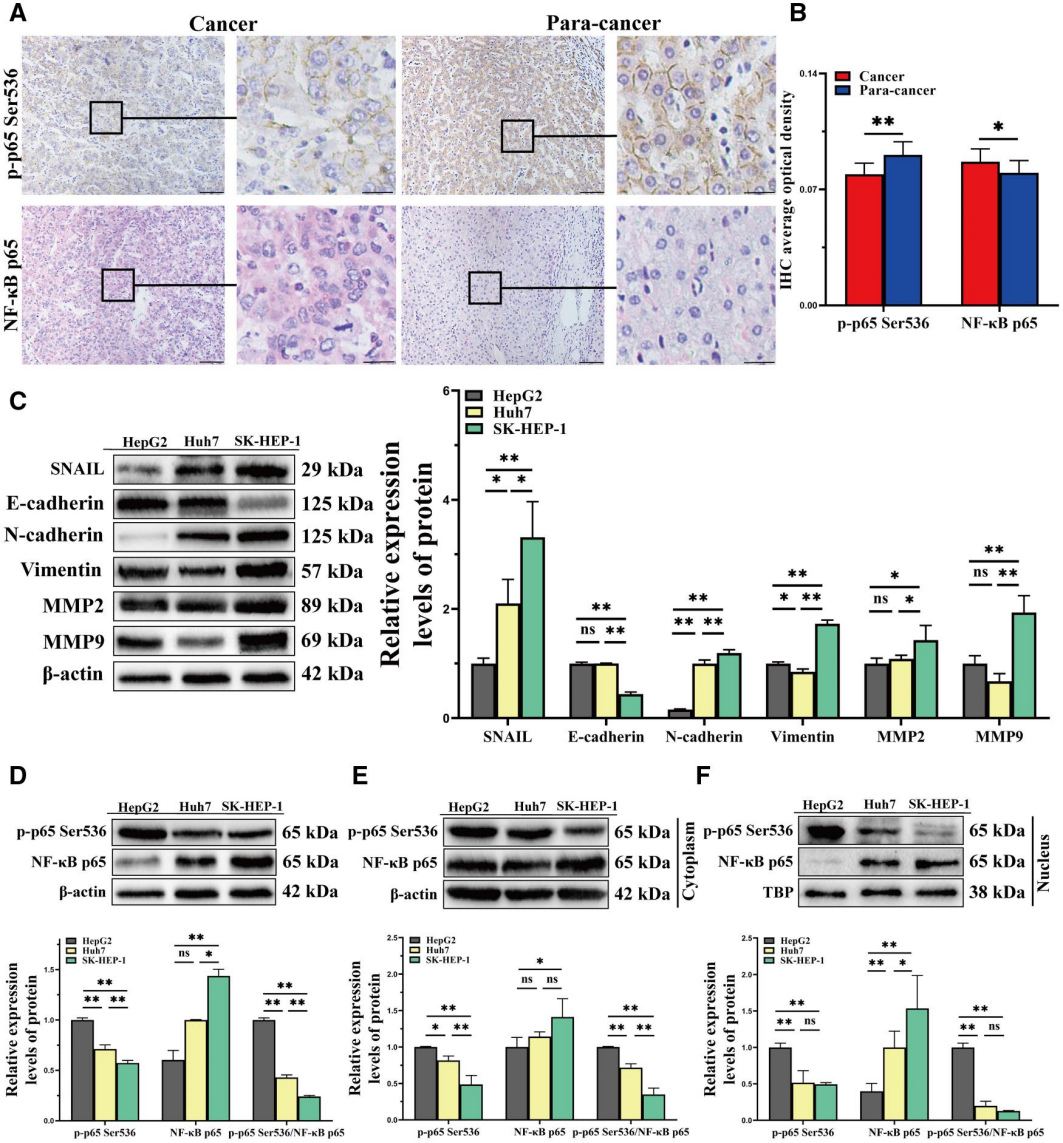
Expressions of p-p65 Ser536 and NF-κB p65 in HCC tissues and cells. (A) Representative immunohistochemical images for HCC tissues and paired para-cancerous tissues (scale bar: 25 μm). (B) Average optical density scores for HCC tissues and paired para-cancerous tissues. (C) Relative protein expressions of *SNAIL*, E-cadherin, N-cadherin, vimentin, *MMP2*, and *MMP9* were detected via Western blotting. (D–F) p-p65 Ser536 and NF-κB p65 in total, the cytoplasm, and the nucleus were examined by using Western blotting. The data shown are the results of three independent experiments and are shown as means ± SD. n.s. = no significance. *P <* *0.05; **P <* *0.01. HCC = hepatocellular carcinoma, IHC = immunohistochemistry, SD = standard deviation.

We first investigated the expression of p-p65 Ser536 and NF-κB p65 in the tissue samples. We found an interesting phenomenon in tissue samples from 12 patients with intermediate and advanced HCC. p-p65 Ser536 expression was lower and NF-κB p65 expression was higher in HCC tissues than in para-cancerous tissues (Figure 1A and B). To explore the correlation between p-p65 Ser536 and the degree of HCC malignancy, we selected three HCC cell lines (HepG2, Huh7, and SK-HEP-1) for further validation. NF-κB p65 increased as HCC malignancy increased, while p-p65 Ser536 decreased (Figure 1C–F). The above results suggest that RelA/p65 Ser536 phosphorylation is not well expressed in intermediate and advanced HCC, and is negatively associated with HCC migration and metastasis.

### RelA/p65 Ser536 phosphorylation suppresses HCC metastasis *in vivo*

**Figure 2.**
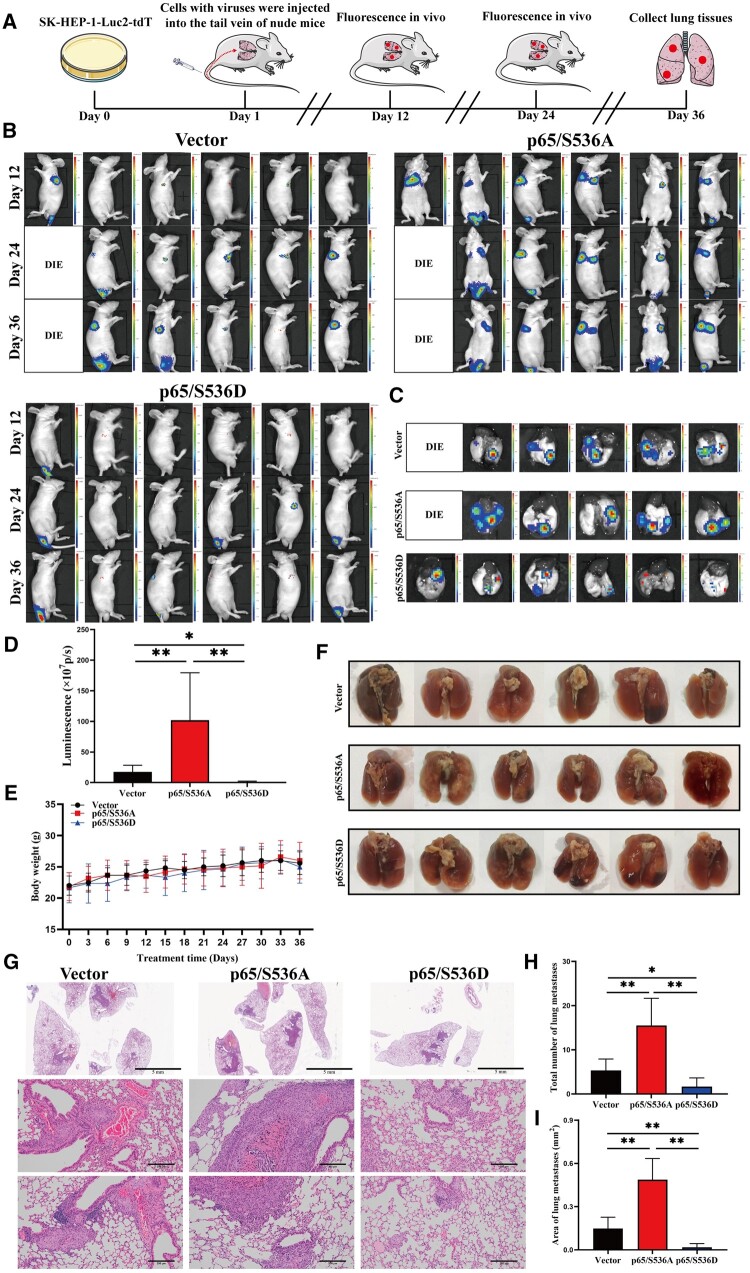
RelA/p65 Ser536 phosphorylation inhibits tumor metastasis. (A) Diagram of the lung metastasis model. (B) Representative bioluminescence images of nude mice. (C) Representative bioluminescence images of the lungs from the nude mice. (D) Fluorescein intensity of the lungs of the nude mice in each group. (E) Body weights of the nude mice. (F) Gross observations of lung metastases. (G) Representative images of intrahepatic metastasis (scale bar: 200 μm). (H, I) Area and total numbers of lung metastases. The data shown are the means ± SD. *P <* *0.05; **P <* *0.01. SD = standard deviation.

We established a mouse model of HCC cell lung metastasis by using tail vein injection of SK-HEP-1-Luc2-tdT cells with AAV-vector, AAV-p65/S536A, or AAV-p65/S536D plasmid solution to validate the effect of RelA/p65 Ser536 phosphorylation on tumor metastasis (Figure 2A). Tumor luciferase intensity was monitored to estimate the tumor volume in nude mice by using an optical imaging system every 12 days (Figure 2B). Lungs were harvested and examined for the luciferase intensity of metastatic tumor nodules after 36 days (Figure 2C). The fluorescence intensity of metastatic lung nodules in the p65/S536D group was lower than that in the vector and p65/S536A groups. For the vector group, metastatic lung nodules in the p65/S536A group of nude mice exhibited a stronger fluorescence intensity (Figure 2D). There was no distinct difference in body weight between the vector, p65/S536A, and p65/S536D groups (Figure 2E). There were fewer HCC foci in the p65/S536D group than in the vector and p65/S536A groups (Figure 2F). Moreover, there were more HCC foci in the p65/S536A group than in the vector group. Hemotoxylin and eosin staining indicated that the volume and number of metastatic lung nodules in the p65/S536D group were lower than those in the vector and p65/S536A groups. In comparison, metastatic lung nodules in the p65/S536A group had a larger volume and greater number than those in the vector group (Figure 2G–I). Based on the results, we concluded that RelA/p65 Ser536 phosphorylation clearly inhibits tumor metastasis.

### RelA/p65 Ser536 phosphorylation suppresses HCC progression *in vivo*

**Figure 3.**
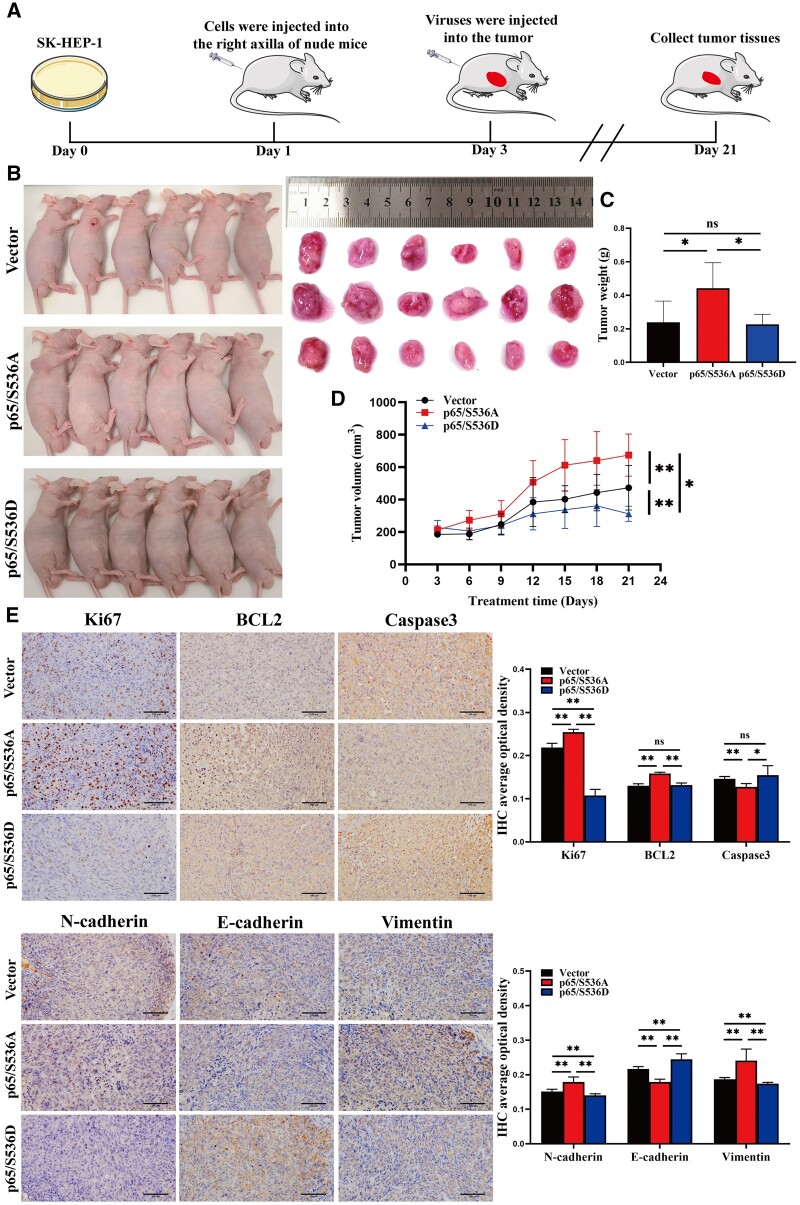
RelA/p65 Ser536 phosphorylation inhibits tumor progression. (A) Diagram of the xenograft models in the nude mice. (B) Representative images of each group of tumor-bearing nude mice and tumors. (C) Weights of tumors in each group of the nude mice. (D) Tumor volume from Days 0 to 21. (E) Representative immunohistochemical staining of Ki67, *BCL2*, Caspase3, N-cadherin, E-cadherin, and vimentin in subcutaneous tumors (scale bar: 100 μm). The data shown are means ± SD. n.s. = no significance. *P <* *0.05; **P <* *0.01. IHC = immunohistochemistry, SD = standard deviation.

The effect of RelA/p65 Ser536 phosphorylation on tumor progression was further confirmed. In SK-HEP-1 cell-bearing nude mice, AAV-vector, AAV-p65/S536A, or AAV-p65/S536D was injected into the tumor (Figure 3A). The nude mice were sacrificed after 21 days. Subcutaneous tumors were collected (Figure 3B). A remarkable reduction in tumor volume in the p65/S536D group was observed compared with the vector and p65/S536A groups, and there was a significant increase in tumor weight and volume in the p65/S536A group compared with the vector group (Figure 3C and D). Histologically, Caspase3 and E-cadherin were upregulated, and Ki67, BCL2, N-cadherin, and vimentin were decreased in the p65/S536D group compared with the p65/S536A group (Figure 3E). Compared with the vector group, Ki67, BCL2, N-cadherin, and vimentin were upregulated, and Caspase3 and E-cadherin were downregulated in p65/S536A-overexpressing subcutaneous tumor tissues. Based on the results from this xenograft model, RelA/p65 Ser536 phosphorylation clearly inhibited tumor progression.

### RelA/p65 Ser536 phosphorylation suppresses NF-κB binding to the *BCL2*, *SNAIL*, and *MMP9* promoters

**Figure 4.**
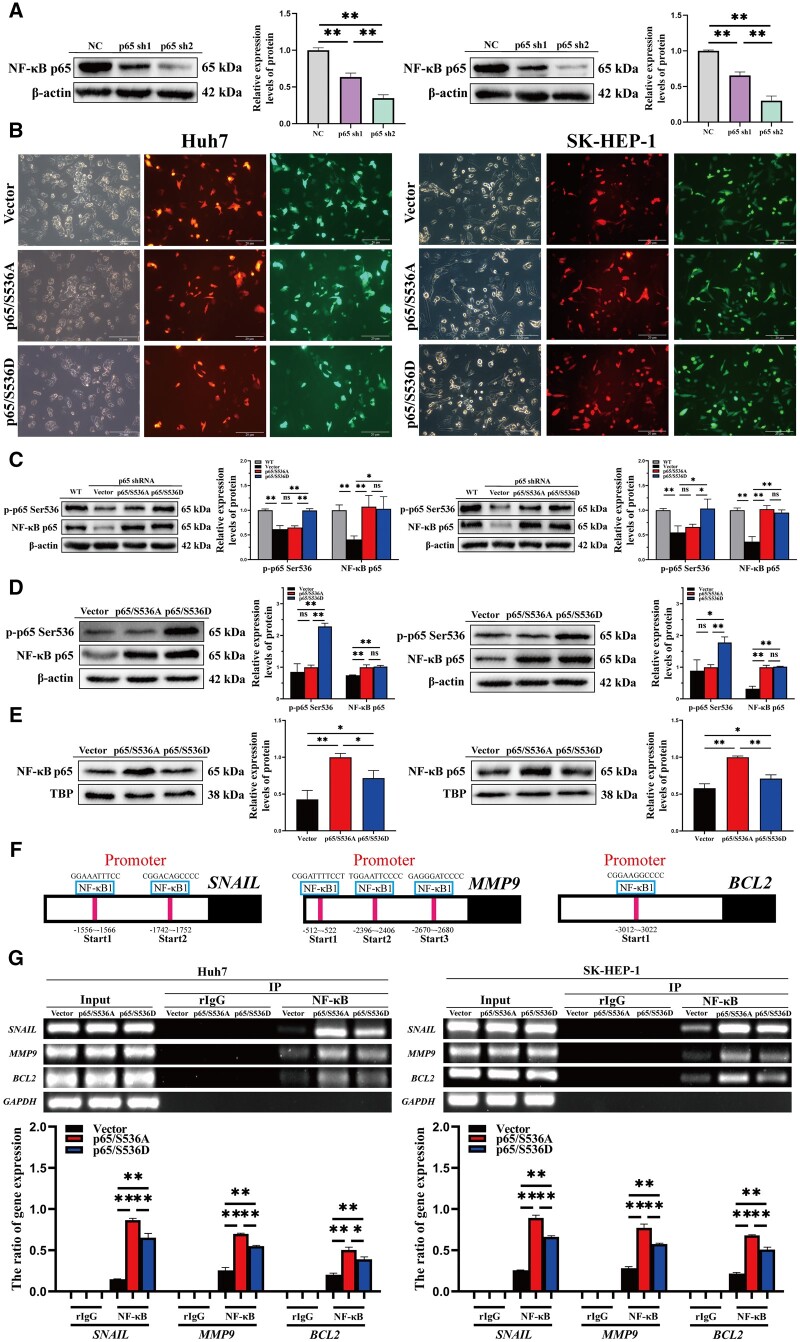
RelA/p65 Ser536 phosphorylation represses *BCL2*, *SNAIL*, and *MMP9* transcription. (A) The expression of endogenous RelA/p65 in HCC cells was detected via Western blotting. (B) Transfection efficiency of plasmids. Representative fluorescent images of HCC cells transfected with the vector, p65/S536A, or p65/S536D after silencing of endogenous RelA/p65. *Middle*: sh-p65, *Right*: vector, p65/S536A, or p65/S536D. (C) The expressions of NF-κB p65 and p-p65 Ser536 were examined via Western blotting. (D) The expressions of NF-κB p65 and p-p65 Ser536 in the cytoplasm were detected by using Western blotting. (E) The expression of NF-κB p65 in the nucleus was detected by using Western blotting. (F) Predicted binding sites for *NF-κB1* and *SNAIL*, *BCL2*, and *MMP9* promoters. (G) The relationship between NF-κB p65 and the promoter regions of *SNAIL*, *BCL2*, and *MMP9* in HCC cells was detected via ChIP. The data shown are the results of three independent experiments and are shown as means ± SD. n.s. = no significance. *P <* *0.05; **P <* *0.01. HCC = hepatocellular carcinoma, sh = short hairpin, WT = wild type, ChIP = chromatin immunoprecipitation, SD = standard deviation.

To avoid endogenous NF-κB p65 interference affecting the accuracy of the experimental results, we used p65 shRNA to silence endogenous RelA/p65 and p65 sh2 was chosen for further experiments (Figure 4A). p65/S536A or p65/S536D was subsequently introduced into the cells by using electro-transfection to reconstitute RelA/p65 (Figure 4B). p65/S536D mimics the phosphorylation of RelA/p65 Ser536 and can be detected by using anti-p-p65 Ser536 antibody, whereas p65/S536A is phosphorylation-deficient and cannot be detected by using anti-p-p65 Ser536 antibody. As depicted in Figure 4C, these two mutants mimic the deletion and phosphorylation of RelA/p65 Ser536.

Previous studies have revealed that RelA/p65 Ser536 phosphorylation affects NF-κB p65 nuclear translocation in T cells [23, 24]. We also observed the same phenomenon by detecting NF-κB p65 content in the cytoplasm and nucleus. The content of NF-κB p65 in the nucleus of HCC cells transfected with p65/S536D was significantly reduced compared with cells transfected with p65/S536A (Figure 4D and E). The binding of NF-κB to target genes such as *BCL2*, *SNAIL*, and *MMP9* is directly affected by the accumulation of NF-κB in the nucleus [25–27]. Binding sites of *NF-κB1* to promoter regions of *BCL2*, *SNAIL*, and *MMP9* genes were predicted from the hTFtarget database (http://bioinfo.life.hust.edu.cn/hTFtarget#!/) (Figure 4F). Chromatin immunoprecipitation assays showed that the binding of *NF-κB1* to *BCL2*, *SNAIL*, and *MMP9* promoters was significantly reduced in p65/S536D-overexpressing HCC cells (Figure 4G). The above experiments suggested that RelA/p65 Ser536 phosphorylation inhibited the nuclear translocation of NF-κB p65 and reduced the transcription of *BCL2*, *SNAIL*, and *MMP9.*

### RelA/p65 Ser536 phosphorylation promotes HCC cell apoptosis

**Figure 5.**
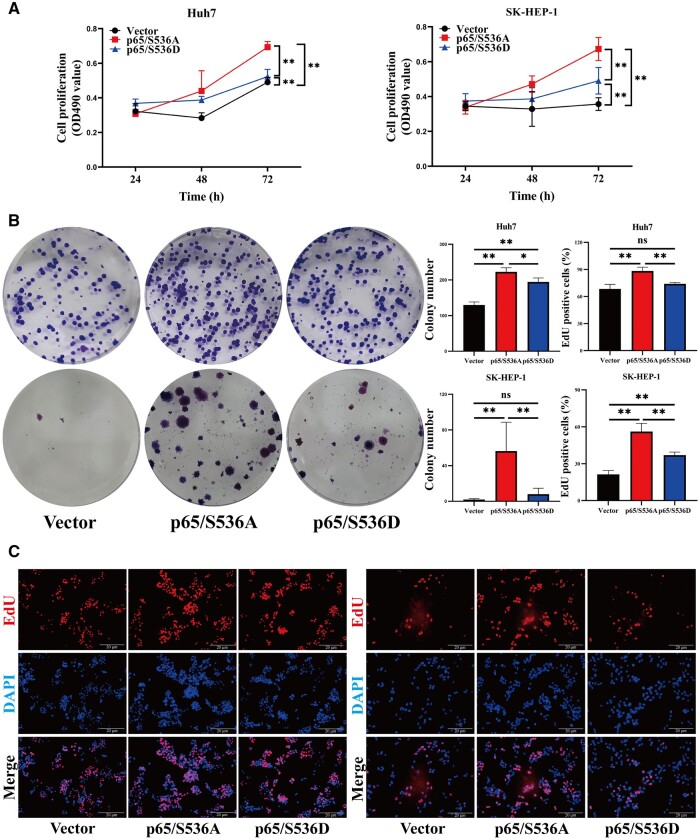
RelA/p65 Ser536 phosphorylation suppresses HCC cell proliferation. (A–C) The proliferation ability of HCC cells transfected with the vector, p65/S536A, and p65/S536D was detected by MTT, colony formation, and EdU incorporation assays (scale bar: 20 μm). The data shown are the results of three independent experiments and are shown as means ± SD. n.s. = no significance. *P <* *0.05; **P <* *0.01. HCC = hepatocellular carcinoma, MTT = tetrazolium-based colorimetric, SD = standard deviation.

**Figure 6.**
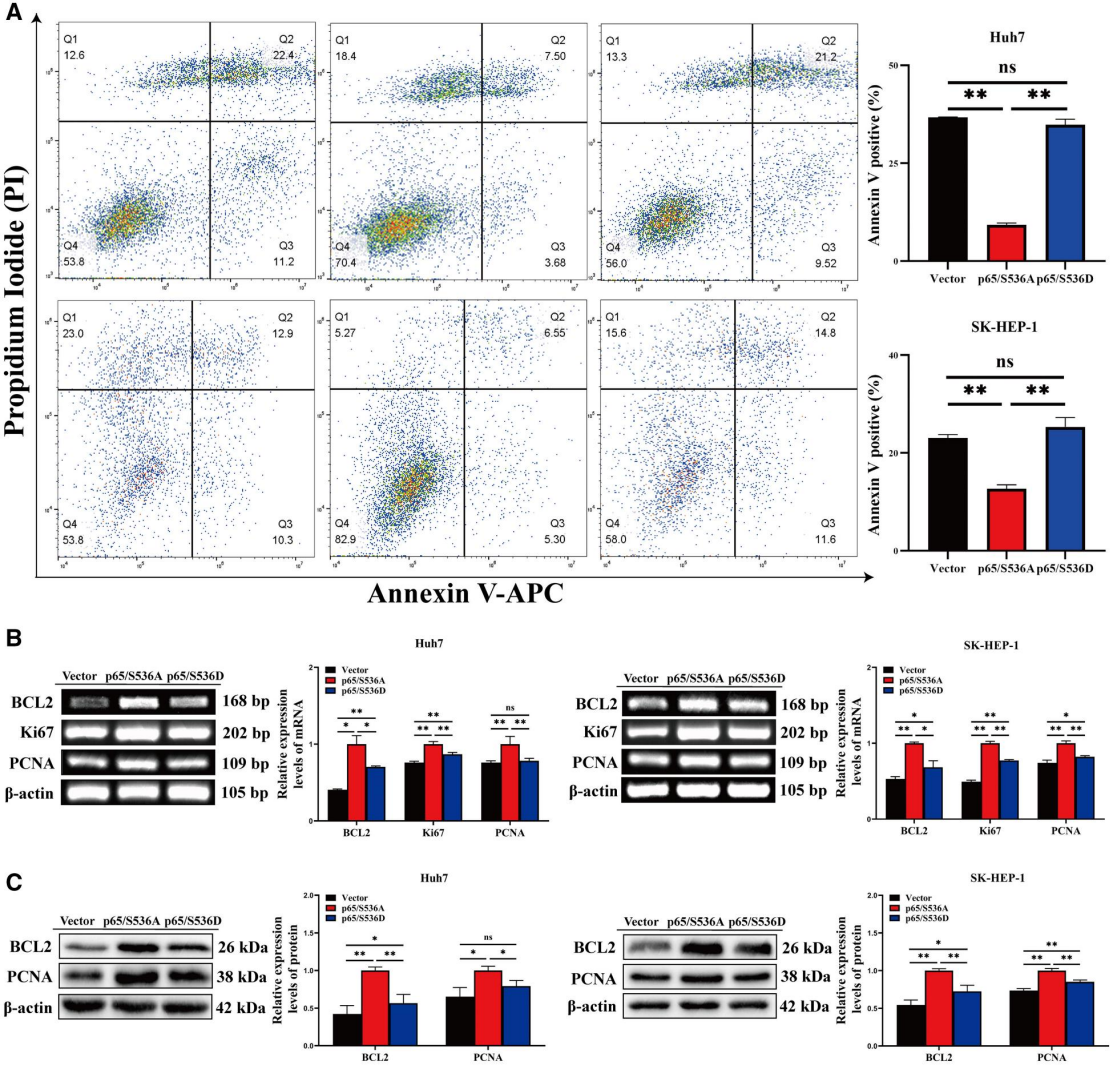
RelA/p65 Ser536 phosphorylation promotes HCC cell apoptosis. (A) The apoptosis rate of HCC cells transfected with the vector, p65/S536A, and p65/S536D was determined via flow cytometry. (B) Relative mRNA levels of *BCL2*, Ki67, and PCNA were determined by using RT–PCR. (C) Relative protein expressions of *BCL2* and PCNA were determined via Western blotting. The data shown are the results of three independent experiments and are shown as means ± SD. n.s. = no significance. *P <* *0.05; **P <* *0.01. HCC = hepatocellular carcinoma, SD = standard deviation.

As mentioned above, the experimental results for chromatin immunoprecipitation showed that p65/S536D reduced *BCL2*, *SNAIL*, and *MMP9* transcription. Subsequently, we explored the biological effects of RelA/p65 Ser536 phosphorylation on HCC cells. First, MTT, colony formation, and EdU incorporation assays were used to study the proliferation and apoptosis of HCC cells. Growth curves demonstrated that p65/S536D-overexpressing HCC cells had lower cell viability compared with p65/S536A-overexpressing cells (Figure 5A). Similarly, p65/S536D-overexpressing HCC cells had a lower number of cell clones in the colony assay (Figure 5B). EdU incorporation assay further demonstrated that the proportions of EdU-positive cells in Huh7 and SK-HEP-1 cells in the p65/S536A group accounted for 88.3% and 56.1%. In contrast, the percentages of EdU-positive cells in Huh7 and SK-HEP-1 cells in the p65/S536D group were 73.9% and 36.9% (Figure 5C). In comparison to HCC cells transfected with p65/S536A, the proportion of EdU-positive cells was markedly decreased in cells transfected with p65/S536D. After 48 h of transfection, the apoptosis rate of Huh7 and SK-HEP-1 cells with overexpressed p65/S536D increased by ∼20.44% and ∼12.64% compared with Huh7 and SK-HEP-1 cells with overexpressed p65/S536A (Figure 6A). The expression of proliferation- and apoptosis-related genes was further examined by using RT–PCR and Western blotting. The RT–PCR results demonstrated that the expressions of anti-apoptotic factors *BCL2*, Ki67, and proliferating cell nuclear antigen (PCNA) were significantly suppressed in p65/S536D-overexpressing HCC cells compared with p65/S536A-overexpressing HCC cells (Figure 6B). Simultaneously, downregulation of *BCL2* and PCNA was found in HCC cells transfected with p65/S536D by using Western blotting (Figure 6C). The above experiments suggested that RelA/p65 Ser536 phosphorylation promotes apoptosis by downregulating *BCL2*, Ki67, and PCNA.

### RelA/p65 Ser536 phosphorylation induces HCC cell cycle arrest

**Figure 7.**
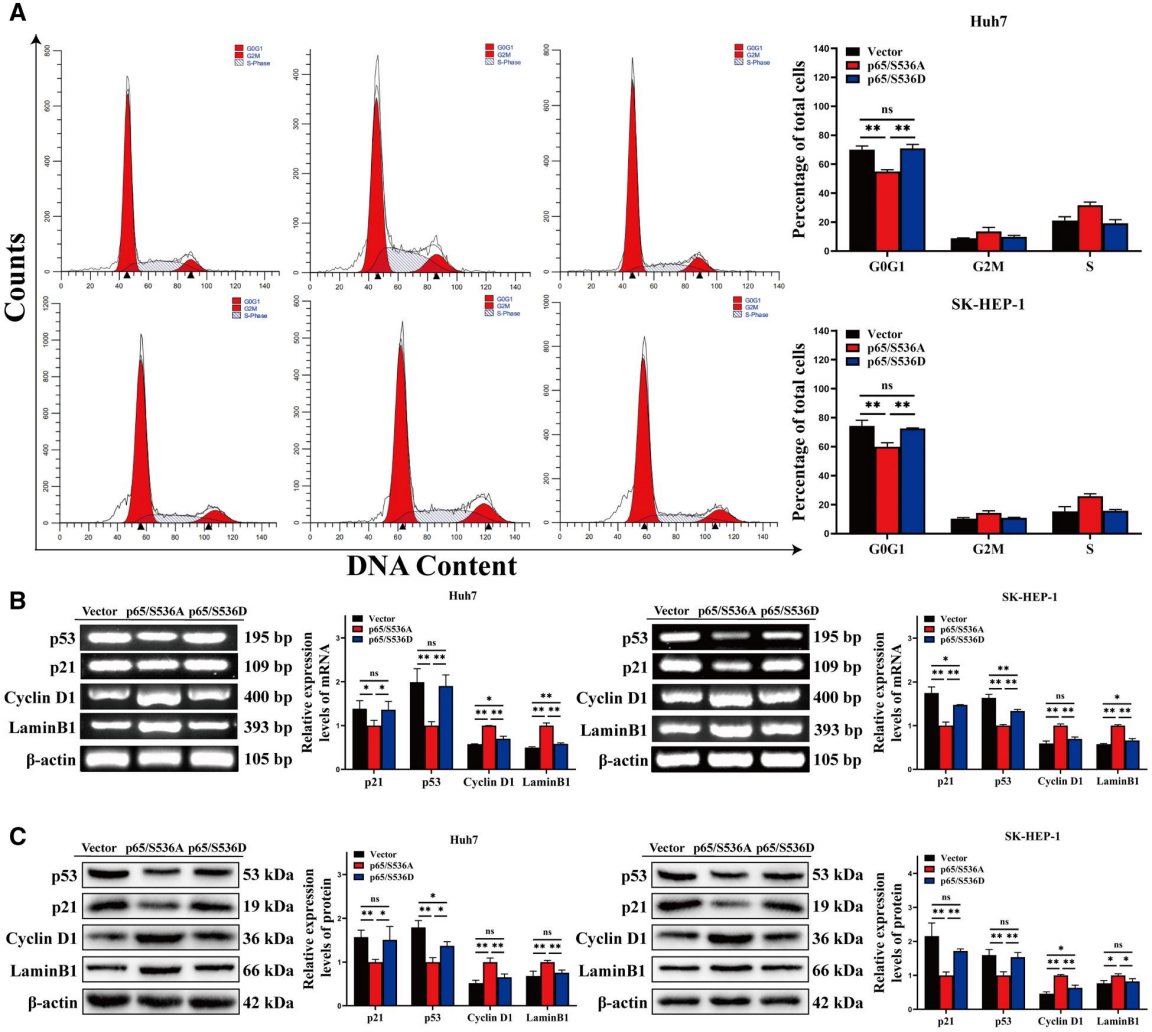
RelA/p65 Ser536 phosphorylation induces HCC cell cycle arrest. (A) The cell cycle profile of HCC cells transfected with vector, p65/S536A, and p65/S536D was determined by using flow cytometry via Annexin V-APC/PI staining. (B) Relative mRNA levels of p21, p53, Cyclin D1, and LaminB1 were determined via RT–PCR. (C) Relative protein expressions of p21, p53, Cyclin D1, and LaminB1 were determined via Western blotting. The data shown are the results of three independent experiments and are shown as means ± SD. n.s. = no significance. *P <* *0.05; **P <* *0.01. HCC = hepatocellular carcinoma, SD = standard deviation.

We further explored the effect of RelA/p65 Ser536 phosphorylation on the cell cycle of HCC cells. Flow cytometry results indicated that the proportions of the G_0_/G_1_ phase in SK-HEP-1 and Huh7 cells with overexpressed p65/S536A were 54.90% and 59.94%, respectively. The percentages of G_0_/G_1_ phase in SK-HEP-1 and Huh7 cells with overexpressed p65/S536D were 70.97% and 72.56% (Figure 7A). Thus, G_0_/G_1_ phase cycle arrest occurred in HCC cells transfected with p65/S536D. Additionally, upregulation of p21 and p53 and downregulation of Cyclin D1 and LaminB1 were found in p65/S536D-overexpressing HCC cells compared with p65/S536A-overexpressing cells via RT–PCR (Figure 7B). Similarly, p21 and p53 were promoted. Cyclin D1 and LaminB1 were suppressed in HCC cells transfected with p65/S536D according to Western blotting (Figure 7C). These data suggested that RelA/p65 Ser536 phosphorylation induces cycle arrest by upregulating p21 and p53, and downregulating Cyclin D1 and LaminB1.

### RelA/p65 Ser536 phosphorylation suppresses HCC cell migration and invasion

**Figure 8.**
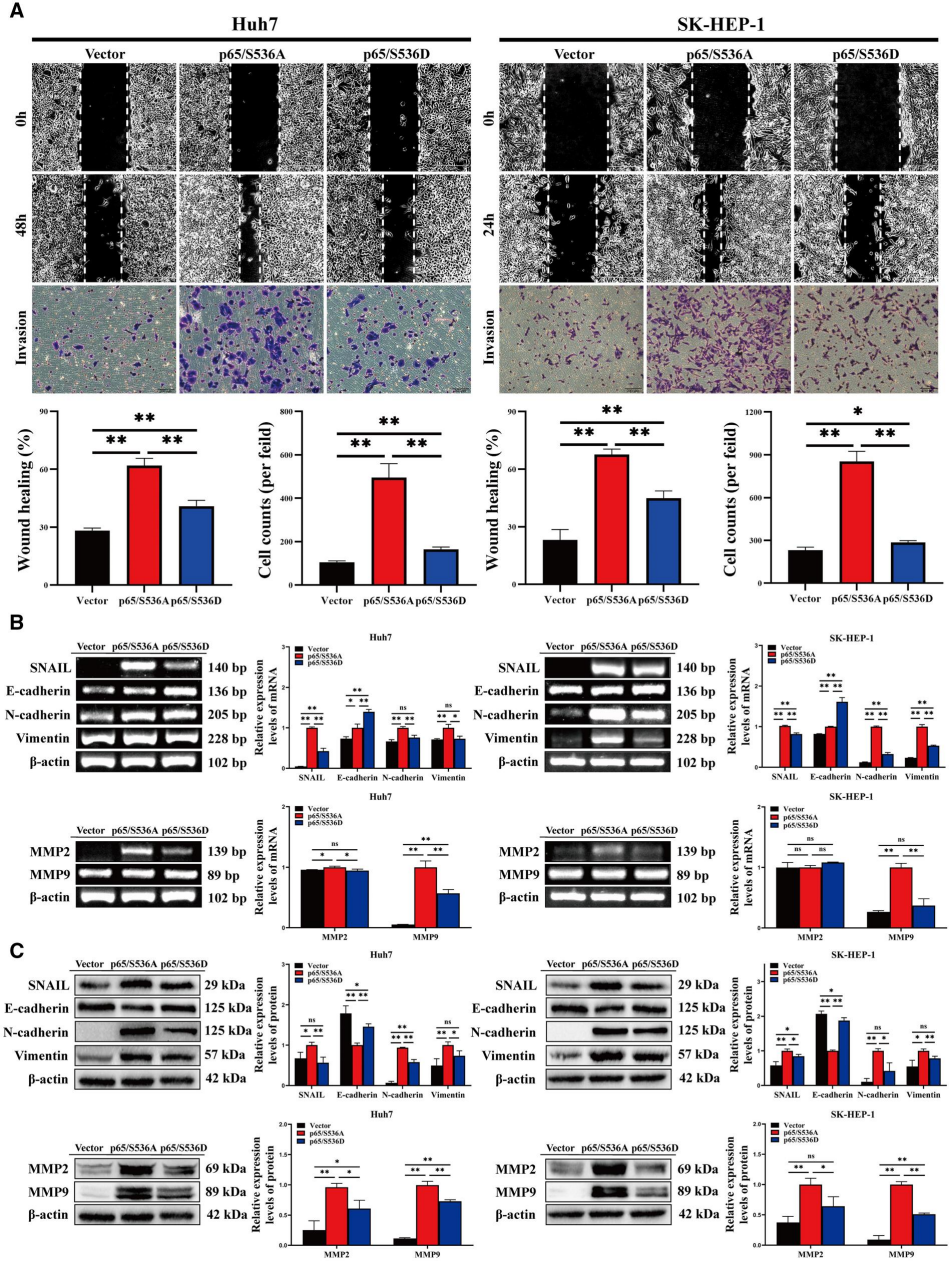
RelA/p65 Ser536 phosphorylation inhibits HCC cell migration and invasion. (A) The migration and invasion abilities of HCC cells transfected with vector, p65/S536A, and p65/S536D were detected via a cellular wound-healing assay and transwell assay (scale bar: 200 μm). (B) Relative mRNA levels of *SNAIL*, E-cadherin, N-cadherin, vimentin, *MMP2*, and *MMP9* were determined by using RT–PCR. (C) Relative protein expressions of *SNAIL*, E-cadherin, N-cadherin, vimentin, *MMP2*, and *MMP9* were determined via Western blotting. The data shown are the results of three independent experiments and are shown as means ± SD. n.s. = no significance. *P <* *0.05; **P <* *0.01. HCC = hepatocellular carcinoma, SD = standard deviation.

Finally, we assessed the effect of RelA/p65 Ser536 phosphorylation on HCC cell migration and invasion. The cellular wound-healing assays results showed that the wound-healing rates were 61.90% and 67.60% in Huh7 and SK-HEP-1 cells with overexpressed p65/S536A and 40.91% and 44.95% in Huh7 and SK-HEP-1 cells with overexpressed p65/S536D. The invasion assays results showed that the numbers of cells that crossed the membrane were 452.4 and 852.7 in HCC cells transfected with p65/S536A, and 156.2 and 285.0 in cells transfected with p65/S536D (Figure 8A). The above results illustrated that the migration and invasion abilities of HCC cells transfected with p65/S536D were inhibited. In comparison with p65/S536A-overexpressing HCC cells, E-cadherin was significantly upregulated in p65/S536D-overexpressing cells, whereas *SNAIL*, N-cadherin, vimentin, *MMP2*, and *MMP9* were significantly downregulated (Figure 8B and C).

## Discussion

As a critical nuclear transcription factor, NF-κB regulates hundreds of genes, including those for cell proliferation and metastasis, and its activation plays a crucial role in HCC development and metastasis [3, 28]. In a previous study, we found that, in 76 pairs of HCC tissue samples, p-p65 Ser536 was significantly under-expressed in advanced HCC, which was negatively correlated with the degree of infiltration of HCC [29]. Further analysis showed that NF-κB p65 was highly expressed in the intermediate and advanced HCC tissues, while p-p65 Ser536 was low. In para-cancerous tissues, NF-κB p65 was low, while p-p65 Ser536 was high. Immunohistochemical analysis of tissue samples revealed the novel finding of downregulation of p-p65 Ser536 expression in intermediate and advanced HCC, in contrast to its high expression in the early stages of acute inflammation-induced HCC [17]. It has been demonstrated that, in HCC that is caused by carcinogens such as DEN, NF-κB is only activated in the acute injury phase to counteract the apoptotic effects of toxicants on the liver. However, in the late stage, NF-κB activation is inhibited to prevent liver damage caused by overactivation [30]. p-p65 Ser536 is highly expressed in DEN-induced HCC, which may be related to the tumorigenic mechanisms behind the different models used. Notably, we found that RelA/p65 Ser536 phosphorylation was under-expressed in intermediate and advanced HCC, and significantly decreased with increasing malignant phenotypes of HCC.

To validate this finding and investigate the corresponding molecular mechanism, we first performed *in vivo* animal experiments. We observed a significant reduction in the volume and weight of p65/S536D-overexpressing tumors. IHC results also showed that Ki67, *BCL2*, N-cadherin, and vimentin were downregulated, and Caspase3 and E-cadherin were upregulated in p65/S536D-overexpressing tumor tissues. To explore specific molecular mechanisms, we silenced cellular endogenous RelA/p65 to exclude interference and then used phosphorylation-deficient mutant p65/S536A and phosphorylation-mimetic mutant p65/S536D to reconstitute the RelA/p65 expression. By separating the cytoplasmic and nuclear proteins, we found that, compared with p65/S536A-overexpressing HCC cells, the entry of NF-κB p65 into the nucleus was significantly reduced in p65/S536D-overexpressing cells, which is consistent with previous studies on NF-κB p65 nuclear dynamics [24, 31]. Overexpression of p65/S536D in HCC cells promotes apoptosis and induces cell cycle arrest. NF-κB acts as a nuclear transcription factor, entering the nucleus to bind to the *BCL2* promoter and regulate apoptosis [32, 33]. Upon further exploration of the mechanism, we found that the binding of NF-κB p65 to the *BCL2* promoter was reduced in p65/S536D-overexpressing HCC cells. *BCL2*, PCNA, Ki67, Cyclin D1, and LaminB1 expressions were inhibited, and p21 and p53 expressions were promoted in HCC cells transfected with p65/S536D. The above study further demonstrated that RelA/p65 Ser536 phosphorylation blocks NF-κB p65 nuclear translocation and inhibits NF-κB p65 binding to the *BCL2* promoter, leading to HCC apoptosis and cycle arrest to suppress tumor growth.

Apoptosis affects not only tumor growth, but also tumor metastasis. *BCL2* plays a critical role in anti-apoptosis and several studies have shown that downregulation of *BCL2* leads to decreased tumorigenicity and metastatic potential [34–36]. Cancer cells release matrix metalloproteinases (MMPs) during tumor metastasis to dissolve the extracellular matrix, enter the circulatory system, metastasize, and colonize distant sites. Additionally, this process relies on EMT [37, 38]. In this process, as epithelial cells, hepatocytes lose their epithelial properties and become mesenchymal cells with mobility. The NF-κB pathway is activated, and migration and invasion are enhanced [39–41]. *SNAIL* is a crucial transcription factor that suppresses E-cadherin and promotes EMT and HCC development [42]. The effect of RelA/p65 Ser536 phosphorylation on tumor metastasis was further explored. We found that the number and tumor area of lung metastatic nodules were reduced in the mice that were injected with p65/S536D-overexpressing HCC cells. Cell migration and invasion were decreased; the expressions of EMT markers, *MMP2*, and *MMP9* were suppressed in p65/S536D-overexpressing HCC cells; and binding of NF-κB p65 to *SNAIL* and *MMP9* promoters was reduced. Thus, RelA/p65 Ser536 phosphorylation enables the blocking of NF-κB p65 nuclear translocation and inhibits NF-κB p65 binding to the *SNAIL* and *MMP9* promoters. It also downregulates the EMT markers, *MMP2* and *MMP9*, and inhibits migration and invasion of HCC.

This study provides insight into the molecular mechanisms by which RelA/p65 Ser536 phosphorylation inhibits the progression and metastasis of intermediate and advanced HCC. In this study, the removal of endogenous RelA/p65 interference followed by the introduction of RelA/p65 Ser536 phosphorylation provided a more realistic view of the role of RelA/p65 Ser536 phosphorylation on HCC progression and metastasis. Metastatic growth of cancer cells largely depends on the role of EMT and seeding of tumor stem cells is the initial prerequisite for metastatic growth [43]. p65/S536D not only promoted apoptosis of HCC cells, but also inhibited migration and invasive ability, which work together to inhibit tumor metastasis. Hundreds of different NF-κB inhibitors have been used to explore cancer treatment, but their clinical application has failed to achieve the expected results [44, 45]. This may be because phosphorylation of the RelA/p65 site is expressed differently in HCC caused by different etiologies and stages of HCC, and there is no selective application of NF-κB inhibitors. We found for the first time that RelA/p65 Ser536 phosphorylation can inhibit HCC development and metastasis, and it is a novel target for the treatment of intermediate and advanced HCC. However, RelA/p65 at the Ser536 site can be phosphorylated by different kinases, including IKKα, IKKβ, IKKε, Ribosomal Subunit S6 Kinase 1, NF-κB-activated kinase, and TANK-binding kinase 1 [31, 46, 47]. Because different kinases can activate the same site and the same kinase can promote protein modification at multiple sites, several compounds that target kinases of NF-κB regulatory factors have been developed [11, 48]. However, kinases that target RelA/p65 at the Ser536 site without affecting other sites still need further investigation.

Despite our findings, our study had some limitations. First, in this study, our group collected a small number of tissue samples from HCC patients and did not collect the clinical data of those patients. Thus, we did not explore the correlation between RelA/p65 Ser536 phosphorylation and clinicopathological characteristics. Second, in animal experiments, endogenous RelA/p65 was not knocked out in nude mice, which may have led to some potential bias.

## Conclusion

**Figure 9.**
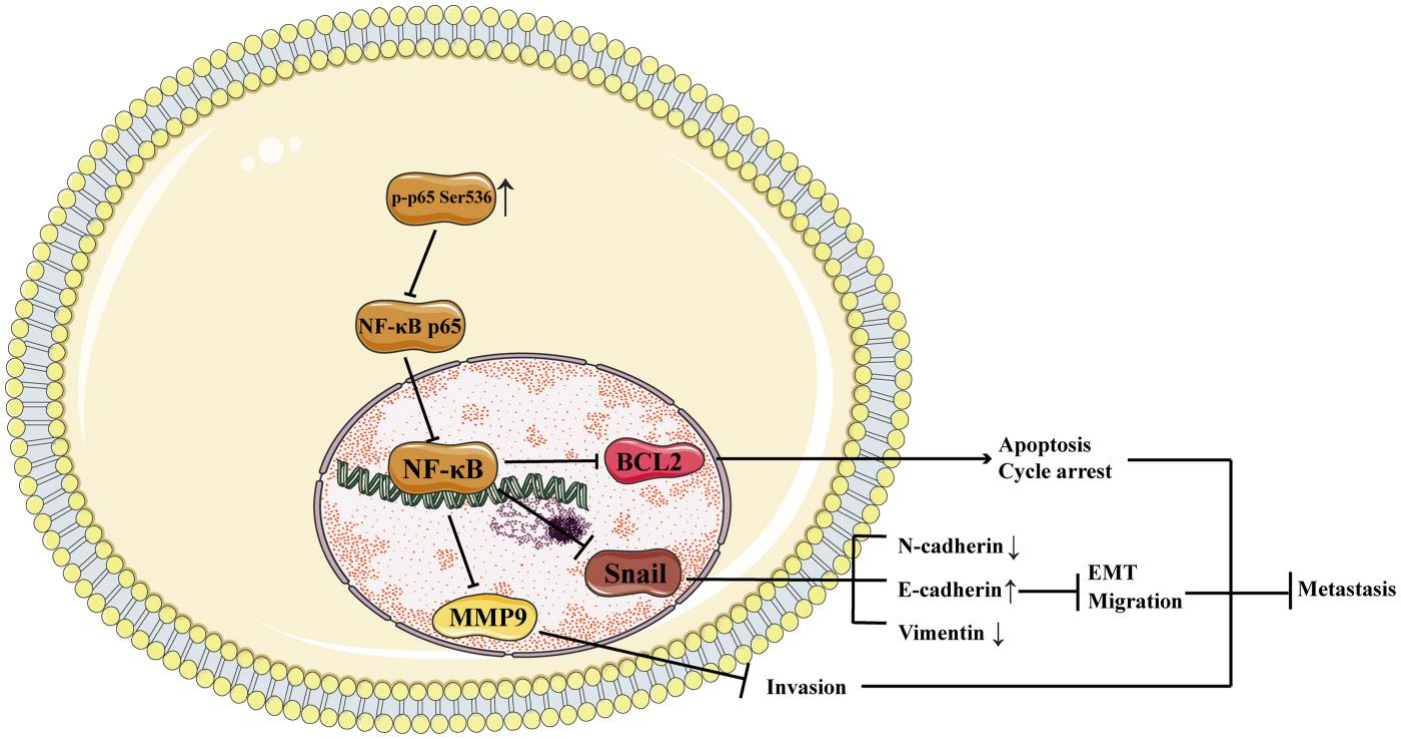
Schematic of the molecular mechanism of the inhibition of progression and metastasis by RelA/p65 Ser536 phosphorylation in HCC

We found low phosphorylation of RelA/p65 Ser536 in HCC, which was negatively correlated with the degree of malignancy of HCC. RelA/p65 Ser536 phosphorylation suppresses HCC progression and metastasis by inhibiting NF-κB p65 nuclear translocation; decreasing NF-κB p65 binding to *BCL2*, *SNAIL*, and *MMP9* promoters; and downregulating *BCL2*, *SNAIL*, and *MMP9* expression (Figure 9). The phosphorylation of the RelA/p65 Ser536 site is a possible target for the treatment of intermediate and advanced HCC. This study will contribute to the development of NF-κB targeted therapies and provide new ideas for the treatment of HCC.

## Supplementary Material

goae094_Supplementary_Data
