# Supplementary material for: Phosphorylation of RelA/p65 Ser536 inhibits the progression and metastasis of hepatocellular carcinoma by mediating cytoplasmic retention of NF-κB p65
Source: Gastroenterol Rep (Oxf). 2024 Nov 4;12:goae094. doi: 10.1093/gastro/goae094 (PMC11534074; doi:10.1093/gastro/goae094)
Supplement: goae094_Supplementary_Data [file goae094_supplementary_data.docx]

**Supplementary Table 1.** p65 shRNA target sequences

| shRNA | Sequence (5’ to 3’) |
| --- | --- |
| p65 sh1 | GGAGCACAGATACCACCAAGA |
| p65 sh2 | GGACATATGAGACCTTCAAGA |

**Supplementary Table 2.** List of antibodies

| Antibody name | Company | Host species | Cat No. | Usage dilution |
| --- | --- | --- | --- | --- |
| NF-κB p65 | Affinity | Rabbit | AF5006 | 1:1000 |
| p-NF-κB p65 (Ser536) | Cell Signaling | Rabbit | 3033 | 1:1000 |
| p-NF-κB p65 (Ser536) | Abcam | Rabbit | ab86299 | 1:200 |
| SNAIL | Affinity | Rabbit | AF6032 | 1:1000 |
| N-cadherin | Abcam | Rabbit | ab76011 | 1:10000 |
| E-cadherin | Abcam | Rabbit | ab40772 | 1:10000 |
| Vimentin | Abcam | Rabbit | ab92547 | 1:5000 |
| MMP9 | Abcam | Rabbit | ab76003 | 1:10000 |
| MMP2 | Abcam | Rabbit | ab92536 | 1:5000 |
| PCNA | Affinity | Rabbit | AF0239 | 1:1000 |
| BCL2 | Beyotime | Mouse | AG1222 | 1:1000 |
| p53 | Abcam | Rabbit | ab32049 | 1:2000 |
| p21 | Abcam | Rabbit | ab109520 | 1:5000 |
| LaminB1 | Proteintech | Rabbit | 12987-1-AP | 1:5000 |
| Cyclin D1 | Beyotime | Rabbit | AF0057 | 1:1000 |
| β-actin | EnoGene | Mouse | E12-041 | 1:1000 |
| TBP | Affinity | Rabbit | AF5476 | 1:1000 |

**Supplementary Table 3.** Primers list for RT**–**PCR

| Primer | Sequence (5’ to 3’) |
| --- | --- |
| SNAIL-F | TCGGAAGCCTAACTACAGCGA |
| SNAIL-R | AGATGAGCATTGGCAGCGAG |
| N-cadherin-F | AGCCAACCTTAACTGAGGAGT |
| N-cadherin-R | GGCAAGTTGATTGGAGGGATG |
| Vimentin-F | GACGCCATCAACACCGAGTT |
| Vimentin-R | CTTTGTCGTTGGTTAGCTGGT |
| E-cadherin-F | ACGTCGTAATCACCACACTGA |
| E-cadherin-R | TTCGTCACTGCTACGTGTAGAA |
| MMP9-F | GGGACGCAGACATCGTCATC |
| MMP9-R | TCGTCATCGTCGAAATGGGC |
| MMP2-F | CCCACTGCGGTTTTCTCGAAT |
| MMP2-R | CAAAGGGGTATCCATCGCCAT |
| PCNA-F | CCTGCTGGGATATTAGCTCCA |
| PCNA-R | CAGCGGTAGGTGTCGAAGC |
| Ki67-F | AGAAGAAGTGGTGCTTCGGAA |
| Ki67-R | AGTTTGCGTGGCCTGTACTAA |
| BCL2-F | ACGGTGGTGGAGGAACTCTTCAG |
| BCL2-R | GGTGTGCAGATGCCGGTTCAG |
| p21-F | CCCGTGAGCGATGGAACT |
| p21-R | CCCGTGGGAAGGTAGAGC |
| p53-F | GTTTCCGTCTGGGCTTCT |
| p53-R | CAACCTCCGTCATGTGCT |
| LaminB1-F | GTTAGCATCTCTCATTCCGCCTCAG |
| LaminB1-R | TGCTTCTTCCTCCTCCTCCTCTTC |
| Cyclin D1-F  Cyclin D1-R | AGGAACAGAAGTGCGAGGAGGAG  ATGGAGGGCGGATTGGAAATGAAC |
| p65-F | ATGTGGAGATCATTGAGCAGC |
| p65-R | CCTGGTCCTGTGTAGCCATT |
| β-actin-F | CACTCTTCCAGCCTTCCTTCC |
| β-actin-R | CGTACAGGTCTTTGCGGATGTC |

**Supplementary Table 4.** Primers list for ChIP assay

| Primer | Sequence (5’ to 3’) |
| --- | --- |
| SNAIL-F | CAGTGATGTGCGTTTCCCTC |
| SNAIL-R | GGACACCTGACCTTCCGAC |
| BCL2-F | TTGGGGTGGCACATCGGCTG |
| BCL2-R | ACTCCGCTGGGTAGTATCTGCTT |
| MMP9-F | AGAGGCTGCTACTGTCCCCT |
| MMP9-R | GGCGGAAGGAATGGGCTCTG |
| GAPDH-F | TACTAGCGGTTTTACGGGCG |
| GAPDH-R | TCGAACAGGAGGAGCAGAGAGCGA |
